# Tunable and scalable production of nanostar particle platforms for diverse applications using an AI-integrated automated synthesis system

**DOI:** 10.1007/s10853-025-10692-1

**Published:** 2025-02-10

**Authors:** Aidan J. Canning, Joy Q. Li, Jianing Chen, Khang Hoang, Taylor Thorsen, Alex Vaziri, Tuan Vo-Dinh

**Affiliations:** a Department of Biomedical Engineering, Duke University, Durham, NC, 27708, USA; b Fitzpatrick Institute for Photonics, Duke University, Durham, NC, 27708, USA; c Department of Chemistry, Duke University, Durham, NC, 27708, USA

**Keywords:** Automation, Synthesis, Nanoparticles, SERS, Photothermal, Scalability

## Abstract

The tunable optical properties and exceptional electromagnetic field enhancement of nanostar-based plasmonic nanoparticles have garnered much attention for use in a wide array of biomedical applications. However, a great challenge for their widespread use is the time-sensitive nature of nanostar synthesis, which could lead to unprecise control of their homogeneity and high batch-to-batch variability. We have developed an automated synthesis system with AI capability to reproducibly synthesize large quantities of nanostar particles. Using this platform, synthesis parameters such as reagent volume and reagent addition timing can be varied to observe how these factors determine the optical properties and SERS enhancement of gold nanostars and bimetallic nanostars, which are used as two model systems. An artificial intelligence (AI) system based on two tree-based machine learning models was developed and trained using nanoparticle characterization data to predict absorbance features and SERS enhancement from synthesis parameters. A grid matrix was fed into the final trained models to create a lookup table to synthesize gold nanostars with an absorbance maximum at specific wavelengths, culminating in the reproducible synthesis of desired nanostar platforms with a peak absorbance wavelength of less than 1.2% difference compared to the target peak absorbance. This machine learning-integrated automated synthesis platform has the potential to enable the next phase of investigation for nanostar-based technologies and expand the scope of their current applications.

## 1. Introduction

Plasmonic nanoparticles have been widely investigated in biomedical applications, from disease biomarker detection to photothermal transduction in cancer therapy [1–3]. This broad applicability stems from optical parameters that can be tuned by altering nanoparticle morphology and elemental makeup [4–6]. These optical parameters are governed by localized surface plasmon resonance, which involves the oscillation of free electrons throughout a nanoparticle at a specific wavelength [7]. Therefore, by altering the morphology and elemental makeup of plasmonic nanoparticles, the localized surface plasmon resonant frequency may be tuned to match commercially available laser sources or biomedically relevant wavelengths. Of all nanoparticle morphologies, gold nanostars (GNS) are of particular interest as their multiple sharp branches create a “lightning rod” effect that enhances local electromagnetic (EM) field dramatically, leading to intense surface-enhanced Raman scattering (SERS) signals. Furthermore, GNS offer the greatest flexibility due to the many features that may be altered during synthesis, including total diameter, number of branches, branch length, branch thickness, and branch tip sharpness [8–16]. Our group first developed a surfactant-free and, therefore, biocompatible GNS synthesis [10]. Since then, several alternative surfactant-free synthetic routes have been developed to produce GNS [12, 17–19]. A biocompatible synthesis for GNS allowed for their investigation as a theranostic photothermal transduction platform to treat solid tumors and be included in multimodal therapies [16, 20–28].

Further, we built upon the surfactant-free GNS synthesis process to create bimetallic nanostars (BNS) [29]. The silver coating on the GNS surface shifts the plasmonic resonant frequency of the particles from the NIR to the visible region, where the intensity of scattered light is greatly increased. The BNS platform has been continuously refined and utilized for the label-free detection of analytes ranging from small molecules to environmental pollutants and biomarkers and acts as a building block for more advanced nanostar-based platforms [30–37]. Furthermore, this resonant frequency blue-shift enables the use of a wide array of commercially available fluorescent dyes in surface-enhanced resonant Raman detection schemes, which can provide an enhancement factor several orders of magnitude higher than SERS alone and is more sensitive for the detection of labeled oligonucleotides compared to fluorescence [38, 39]. Resonant Raman dye-labeled nucleotide probes functionalized to BNS particles have been used to sense circulating cancer miRNA biomarkers, viral infections in plants, and small molecules [40–43].

A great challenge in fabricating nanoparticles is to achieve high reproducibility and precision in their synthesis, which has, to date, limited their use for large-scale implementation. Synthesizing GNS and BNS particles requires rapid and precisely timed addition of several reagents in immediate succession. Varying the addition timing for any single reagent will lead to differences between batches of GNS and BNS particles. Also, the nanostar synthesis reaction is sensitive to differences in reagent concentration throughout the solution mixture when each reagent is added, limiting the volume of each batch and, therefore, often requiring multiple batches for a single experiment. To address the problems associated with batch-to-batch variability, several groups have explored automating the nanostar synthesis developed by our group on microfluidic and milifuidic scales [44–46]. However, these approaches are only used to synthesize GNS particles and either yield a small number of particles or require expensive High-performance liquid chromatography (HPLC) pumps. Other works have integrated complex machine-learning (ML) tools that have also been utilized to aid nanoparticle synthesis by predicting synthesis outcomes or assisting experimental planning [47]. ML models, including Bayesian optimization [48], genetic algorithms [48], and heuristic algorithms [49], have all been used for synthesis optimization. Artificial neural networks (ANNs), gaussian processes, and tree-based models have been previously used to predict nanoparticle synthesis outcomes such as extinction properties and particle size. ML has been shown to aid in the synthesis of metallic nanoparticles, including gold nanoparticles [50, 51], silver nanoparticles [52–54], and multi-metallic nanospheres [55]. ML-controlled synthesis has not yet been reported for gold nanostars and silver-coated nanostars, which have the distinct properties mentioned above that make them highly suitable for photothermal therapies and SERS sensing, respectively.

In this work, we have developed an ML-integrated automated synthesis platform to produce application-specific nanostar particles. This platform is constructed using low-cost, consumer-grade electronics, allowing system flexibility and widespread adoption. An Arduino Mega 2560 microcontroller controls reagent volumes and addition sequences to enable the simple control of the stepper motors, which are used for reagent dispensing. Using this platform, the synthesis of both GNS particles and BNS particles was vigorously investigated by sweeping several parameters, such as the volumes of several reagents and the timing between the addition of reagents. FEM modeling was also used to validate experimental absorbance spectra and SERS enhancement data. Using features derived from nanoparticle batches synthesized using various device parameters, we have developed a two ANN model to predict GNS and BNS synthesis outcomes based on the amount of each reagent and the addition timing sequence. Using these two models, we assembled a look-up table for GNS and BNS synthesis parameters, predicted absorbance features, and relative SERS enhancement. To validate these lookup tables, GNS formulations with predicted peak absorbance values of 808 nm and 1064 nm, two commonly used wavelengths in photothermal therapy, were synthesized with an average percent difference from the target values of 0.04% and 1.15%, respectively. The performance of this automated synthesis system with AI capability is compelling evidence that it has the potential to enable the next phase of investigation for nanostar-based technologies.

## 2. Methods

### 2.1 Synthesis device parts and assembly

All parts used to assemble the automated synthesis platform are commercially available, and the housing is 3D printed. An Arduino 2560 Rev3 microcontroller controls the device used in this study. A maximum of 7 peristaltic pumps by Kamoer are used, each controlled with an A4988 stepper motor driver board. A 25V DC power supply by Korad is used to operate the platform. A 330 uF capacitor is placed in parallel with the motor voltage for each driver board. During operation, the device operates at 25 V while drawing approximately 1 A.

### 2.2 Nanostar synthesis reagents

All reactions mentioned in this work occur in a final volume of 100 mL. A ratio of 1uL of hydrochloric acid to 1mL of deionized water constitutes the dilute hydrochloric acid solution. The concentration of gold chloride solution used in this work is 5.5 mM. A volume of 1mL was used for all reagents unless otherwise stated. The optical density of the gold seed solution used is 2.83 at 520 nm and is prepared via the Turkevich method [56]. The concentration of ascorbic acid used is 100 mM. The concentration of SH-PEG_5000_-COOH used to terminate GNS synthesis was 5 mg/mL. The concentration of the silver nitrate solution used was varied between 1 mM, 2.5 mM, and 5 mM to produce s10, s25, and s50-based nanostars, respectively. The concentration of the silver nitrate solution used to produce BNS particles was 12.5 mM. A 1:10 ammonium hydroxide to DI water solution was used in the final step of BNS particle synthesis.

### 2.3 Nanostar characterization and SERS measurements

All absorbance measurements were recorded using a Shimadzu UV-3600i spectrophotometer over 400– 1200 nm. All HAADF-STEM and EDS-STEM images were taken using an FEI Talos F200X. For each BNS particle formulation, 10 uL of 1 mM p-MBA solution was added to 990 uL of nanoparticle solution and mixed for 20 minutes. Particles were sonicated immediately before SERS measurements to ensure the recorded spectra were generated by monodispersed particles. Measurements were recorded using WP 633 XL Raman spectrometer by Wasatch Photonics, outfitted with a Newton 920 Andor camera. All SERS data displayed is a result of a scanning average of 5 spectra.

### 2.4 Nanostar simulations in COMSOL

COMSOL Multiphysics 6.0 and the wave optic package were used for all FEM simulations performed in this study. The optical constants described by McPeak et al. for gold and silver were used in all simulations [57]. The meshing of the entire domain was physics-dependent and set to extremely fine. The surrounding medium outside the nanoparticle was modeled as water, with the optical properties described by Querry et al. [58]. The s25 model had a core radius of 20 nm, a branch length of 20 nm, the radius of the branch base was 7nm, and the radius of the branch tip was 5 nm. For the s50 model, the core radius is 15 nm, the branch length is 25 nm, the radius of the branch base is 4 nm, and the radius of the branch tip is 3 nm.

### 2.5 Machine learning and data processing

For data processing, absorbance spectra were smoothed with Savitzky-Golay with polynomial order 1 and window length 13. From the smoothed, non-normalized spectrum, the wavelength of the maximum absorbance and absorbance value of the peak were extracted. The absorbance spectrum was then normalized, and the width of the peak at 85% of the normalized maximum was extracted (Fig S1).

Separate models were used to accomplish two distinct tasks. Task 1 was to predict extracted absorbance features from above (peak wavelength, height, and width) using inputs consisting of important experimental parameters from the synthesis machine. The input parameters were dispensed volumes of gold seed (Au), first silver nitrate addition (Ag1), second silver nitrate addition (Ag2), and time between Ag1 and ascorbic acid addition (AA). Task 2 was to predict SERS enhancement at 633nm excitation using, again, inputs as experimental parameters from the synthesis machine: dispensed volume of Au, Ag1, and Ag2. The time between Ag1 and AA was fixed and not used as input for training due to limited dataset. All inputs were normalized to be in the range (0,1) before training. The performance of 5 different ML models, SVR, RF, CatBoost, XGBoost, and ANN, were compared using 5-fold nested cross-validation for each task. Within each outer fold, Optuna with n-=60 trials was used for cross-validation in the inner fold. Parameter ranges are shown in Tables S1–S10. NRMSE was calculated as loss and NRMSE for each outer fold was used to decide the best model. CatBoost and XGBoost performed best in nested CV for tasks 1 and 2, respectively. The tuned hyperparameters in each fold for the selected models were combined in an ensemble model.

The final ensemble, CatBoost, and ensemble XGBoost models were trained on all available data. To produce the ML-generated look-up table of synthesis parameters, absorbance properties, and enhancement properties, a mesh grid of synthesis machine experimental parameters (volumes of Au, Ag1, Ag2, and time between Ag1 and AA) were constructed and used as input. The range of each experimental parameter spanned the training range. The resulting mesh grid was fed into the final ensemble CatBoost and ensemble XGBoost models to predict the absorbance and enhancement properties for each set of experimental parameters for the synthesis machine in the mesh grid. These predictions and parameters are assembled into the look-up table and used to select parameters for the synthesis machine for specific applications.

## 3. Results and Discussion

### 3.1 Automated Synthesis Platform Design

**Figure 1.**
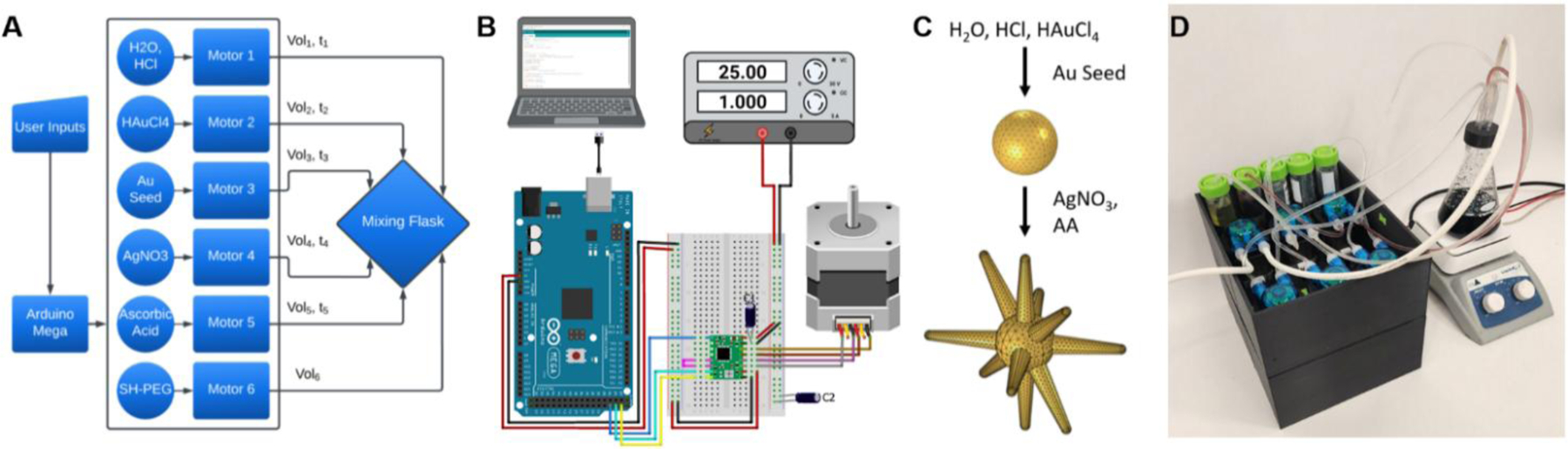
Design of the automated synthesis platform. A) A schematic containing all the reagents and motors used to synthesize GNS particles. B) A diagram showing the connections of a single motor unit. C) Scheme depicting GNS formation. D) Photo of the automated synthesis platform immediately after use.

The active components of the automated synthesis platform are depicted in a schematic diagram (Fig. 1A), where user input is executed by an Arduino Mega 2560 microcontroller, which in turn controls a total of 6 motors that are used to dispense different reagents necessary for GNS synthesis. All volumes and timing between reagent injections are assignable for each run, allowing for the precise control of synthetic conditions. By adjusting these parameters, we can tune the optical properties of the GNS to fit specific application needs. The microcontroller and an A4988 stepper motor driver board control each motor unit (Fig. 1B) of the platform. The stepper motor driver board takes the input commands from the microcontroller and a 25 V potential to operate the motor for reagent dispensing. For a single batch of GNS, 100mL of deionized water pH 3 is added to a rapidly mixing flask. A volume of gold chloride solution is added, followed by 12nm gold nanospheres, silver nitrate, and ascorbic acid. The rapid addition of these four reagents triggers branch formation (Fig. 1C) on the surface of the gold nanospheres, resulting in the GNS morphology. The reaction is then terminated with the addition of Sh-PEG_5000_.

The automated synthesis platform (Fig. 1D) has a two-level design; the top level houses all the stepper motor peristaltic pumps and reagent tubes for direct access. Peristaltic pumps were chosen for incorporation into the device to ensure there was no pump head contamination. The reagent tubes consist of 5 50mL conicals containing gold chloride, gold nanosphere solution, silver nitrate, ascorbic acid, and thiolated PEG solution. A large container containing an acidic aqueous solution is out of view. For all 6 dispensing stepper motor peristaltic pumps, one end of the tubing is placed into the reagent reservoir while the other end is fitted with a pipette tip and inserted into the injection head, which is fitted to the neck of the flask. The injection head ensures no contact between individual injection channels and that all are oriented vertically and directly at the mixing solution below. The lower level houses all the associated circuitry and wiring, offering protection from unintended user contact and reagent solutions (Fig. S2).

### 3.2 Automated Synthesis of Gold Nanostars

**Figure 2.**
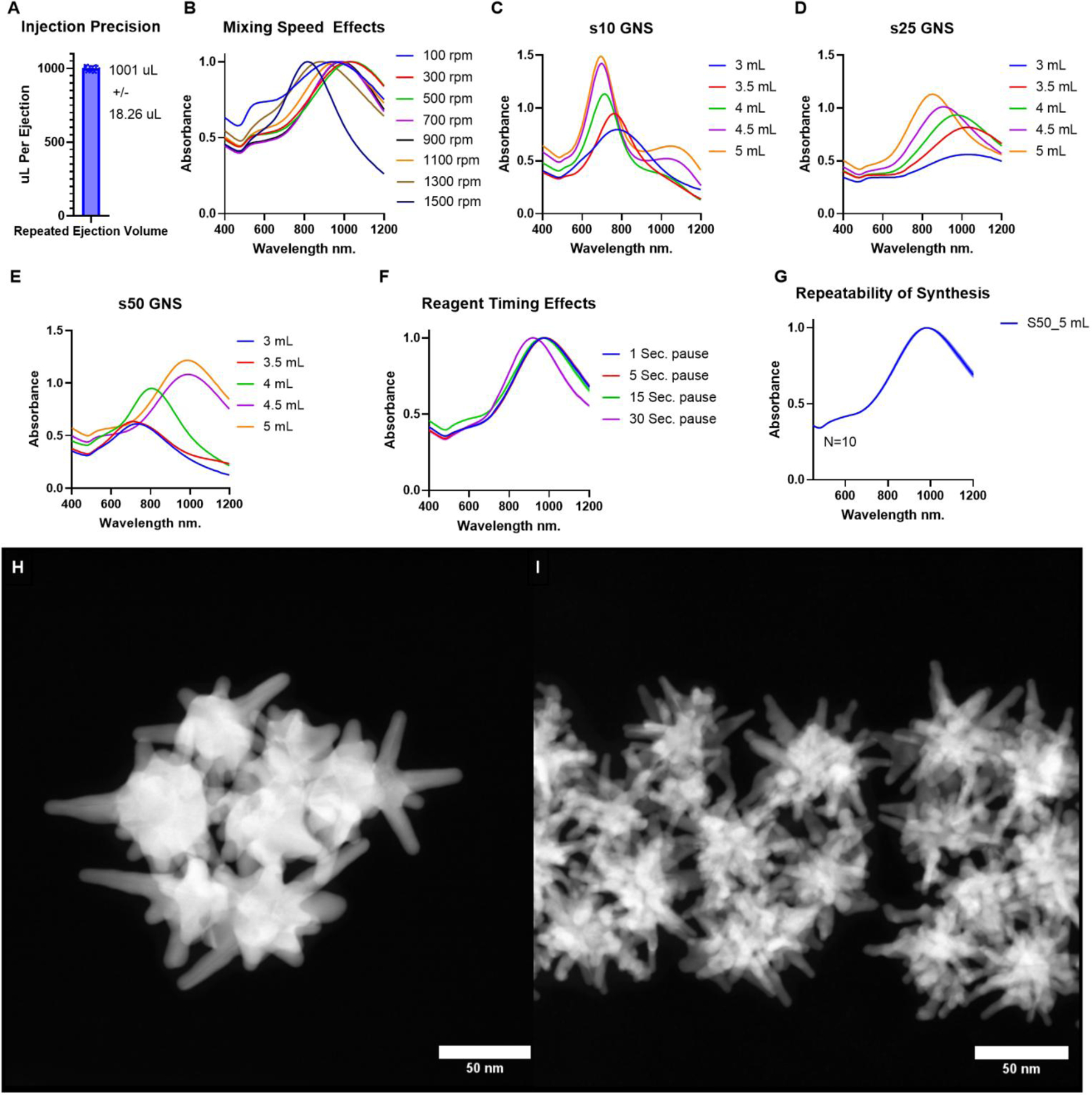
Automated synthesis platform characterization and GNS synthesis A) The volume dispensed 30 times in a row using the GNS synthesis device. B) Absorbance spectra of GNS solutions where the mixing speed was varied. C) Absorbance spectra of base s25 GNS particle as the amount of gold chloride added to the solution was varied. D) Absorbance spectra of base s25 GNS particle as the amount of gold chloride added to the solution was varied. E) Absorbance spectra of base s50 GNS particle as the amount of gold chloride added to the solution was varied. F) The absorbance spectra of base s50 GNS created using the same amount of gold, while varying the delay between silver nitrate and ascorbic acid addition, N=3 for each. G) The absorption spectra of 10 batches using identical parameters, N=10. H) HAADF-STEM image of s25 GNS prepared with 5mL of gold chloride solution. I) HAADF-STEM image of s50 GNS particles prepared with 5mL gold chloride solution.

Several variables were identified during platform development that could significantly impact the final morphology of nanostar particles. These variables included the reaction volume, the quantities of all 6 reagents necessary to achieve stable GNS, the timing between all injection steps, the mixing speed of the solution, and injection volume variability. Therefore, before initiating nanostar synthesis, the injection volume accuracy and precision of the device were measured. A minimum injection volume of 1 mL was selected for this study and was used to characterize injection volume accuracy and precision. The average injection volume after 30 injections in a row was 1001 **μ**L ± 18.26 **μ**L, or 1.8% (Fig. 2A). We then examined the effects of solution mixing speed on nanoparticle synthesis. For initial testing, device settings for reagent amounts and timings were selected based on our experience synthesizing GNS by hand [10]. The reagent addition settings were as follows: 100 mL of the acidic aqueous solution, 1-second pause, 4 ml of gold chloride solution, 1-second pause, 1 mL of gold sphere solution, 2-second pause, 1 mL of 5 mM silver nitrate, 5-second pause, 1 mL of 0.1 M ascorbic acid, 20-second pause, 1 mL of 5 mg/mL SH-PEG_5000_ solution. The mixing speeds tested in this study ranged from 100 rpm to 1500 rpm in steps of 200 rpm. At the lowest speed (Fig. 2B), the absorption peak is the broadest, indicating the formation of highly heterogeneous particles in solution. As the mixing speed increases from 300 to 1300 rpm, the absorbance spectra sharpen, and the peak absorption value blue shifts from 1028 nm to 882 nm. At the highest mixing speed, 1500 rpm, the absorbance spectrum dramatically narrows, and the peak absorption value further blue shifts to 814 nm. This noticeable peak narrowing indicates an increase in the uniformity of GNS particles within the solution relative to other prepared batches. This peak sharpness is desirable in applications such as photothermal therapy or SERS sensing. Based on these findings, a mixing speed of 1500 rpm is used in all future reactions.

Three different base GNS morphologies, labled s10, s25, and s50, where the number refers to the final micromolar concentration of silver nitrate in solution, were investigated to test the tunability of the surfactant-free GNS platform using automated injection volumes and timings. For each of these base morphologies, the amount of gold chloride solution added varied between 3 mL and 5 mL in steps of 0.5 mL. All other volumes and timings were held constant and were the same as what was used in the mixing speed study. Clear trends appear in the absorption spectra (Fig. 2C–2E) for each base GNS morphology. Beginning with s10, as the amount of gold chloride in the solution is increased, the absorbance of the solution increases, the main peak value blue shifts from 782 nm to 696 nm, and the peak narrows. Also, a secondary peak at 1046 nm rises notably when 4.5 mL and 5 mL of gold chloride are added to the solution. A similar trend is also seen in the s25 base morphology; as the amount of gold chloride increases, the peak absorption value increases, and blue shifts from 1030 nm to 850 nm while the peak also narrows. In the s50 base morphology, the peak absorbance value increases as more gold chloride is added to the solution; however, the value redshifts from 730 nm to 988 nm while the peak narrows. The deposition of silver atoms on the spherical gold seed used for initial GNS formation has previously been investigated as the underlying mechanism for shaping the growth of high-aspect structures [8, 59]. Therefore, we suspect that as the amount of silver increases, there is a less continuous area along the seed for gold reduction, resulting in a greater number of high-aspect-ratio branches and a smaller nanoparticle core diameter overall. By altering the amounts of each input reagent, we can effectively span the optical therapeutic window by manipulating the base GNS morphologies with the amount of silver and gold chloride added into the solution.

To examine the effects of altering the amount of time between the addition of silver nitrate, which controls branch thickness, and ascorbic acid, which reduces gold chloride in solution, causing rapid branch formation, 4 different time intervals were tested in triplicate (Fig. 2F). There was little to no difference in the average maximum absorption value for time delays of 1, 5, and 15 seconds, with a slight blue shift occurring at a time delay of 30s. We suspect that the relative insensitivity of the absorption spectrum maximum to changes in this time interval is most likely a result of the extremely rapid mixing speed used in this study. To test the repeatability of the nanoparticle synthesis platform, 10 identical batches of s50 GNS particles prepared with 5mL of gold chloride were synthesized, and the resulting average maximum absorbance value of all 10 batches was 983.8 nm, with a standard deviation of 9.1 nm, or 0.93% (Fig. 2G). To confirm that a noticeable change in particle morphology is the cause of the changes in absorbance spectrum seen as a function of gold chloride and silver nitrate (Fig. 2C–2E), high-angle annular dark field scanning transmission electron microscopy (HAADF-STEM) images of s25 and s50 particles prepared with 5 mL of gold chloride (Fig. 2H–2I). Statistical analysis (Fig. S3A–S3D) of HAADF-STEM images revealed that s25 particles had an average branch length of 23.7 ± 8.6 nm, branch thickness of 9.6 ± 1.4 nm, core diameter of 42.9 ± 5.5 nm, and 8.8 ± 2.1 branches per particle. Whereas s50 particles had an average branch length of 28.8 ± 8.3 nm, branch thickness of 5.1 ± 1.2 nm, core diameter of 28.9 ± 4.2 nm, and 19.7 ± 2.7 branches per particle. The pronounced difference in nanoparticle morphology and resulting optical properties demonstrates the utility of our automated synthesis system.

### 3.3 Theoretical Investigation of Morphological-Dependent Plasmonic Properties

**Figure 3.**
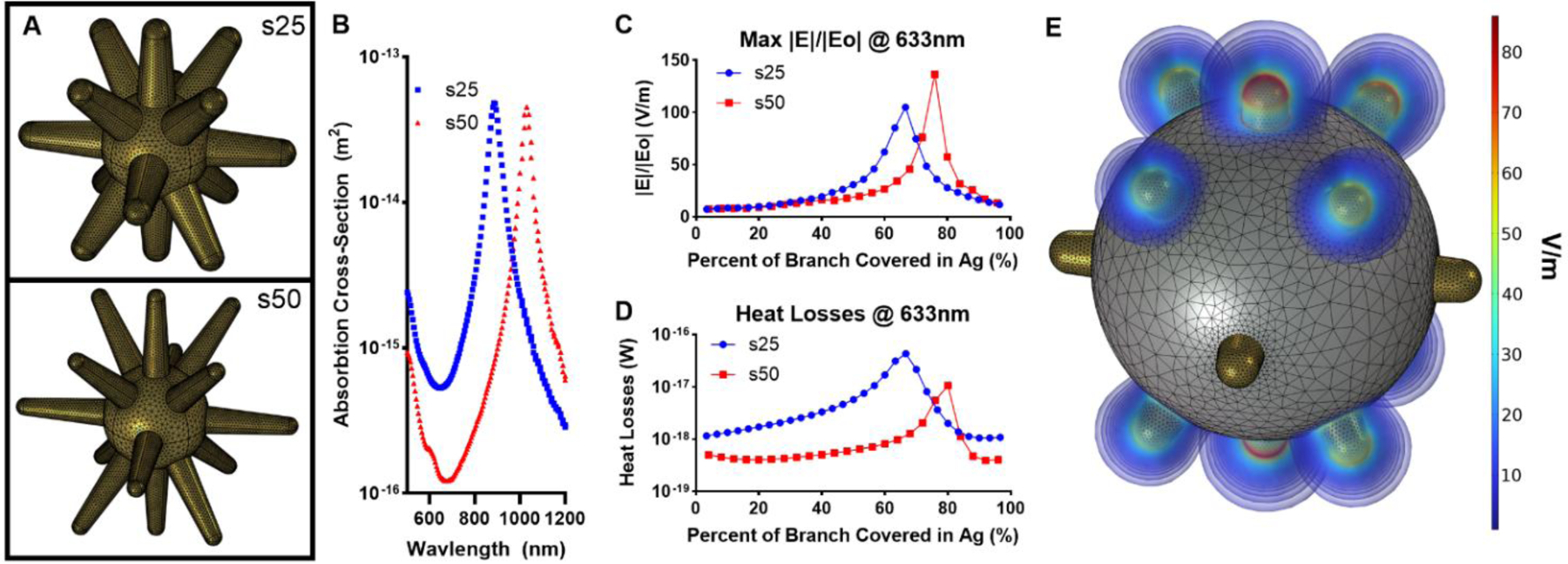
COMSOL investigation of nanostar particles. A) GNS models of s25 and s50 nanostars. B) Simulated absorbance cross sections for s25 and s50 models. C) Maximum electric field enhancement generated at 633 nm. D) Heat losses generated by the nanoparticle model at 633 nm. E) Local electric field around BNS model which had the greatest heat losses value.

COMSOL Multiphysics 6.0 was used in parallel with nanostar synthesis to investigate how nanoparticle morphology changes affected the optical properties of the particles. First, models representing the s25 and s50 GNS morphologies were designed based on STEM images (Fig. 3A). The simulated absorbance cross-section for both models (Fig. 3B) agrees with experimental absorbance spectra (Fig. 2D–2E). The simulated peak absorbance cross-section value for the s25 model occurred at 880 nm, where it occurred at 854 nm experimentally, resulting in a percent difference of 2.95%. Similarly, the simulated peak absorbance cross-section value for the s50 model occurred at 1030 nm and 988 nm experimentally, resulting in a percent difference of 4.08%. This strong agreement between experimental and theoretical results highlights the utility of COMSOL Multiphysics for prediction and validation. Next, the s25 and s50 base models were used to simulate BNS particles with varying silver thicknesses ranging from 1 nm to the end of the branch tips. The maximum electric field enhancement |E|/|Eo| for each model as a function of silver thickness (Fig. 3C) upon 633 nm excitation for the base s25 and s50 BNS particles was 104.8 V/m and 136.3 V/m, respectively. However, the heat losses (Fig. 3D), which considers the electric field across the entire nanoparticle and can more accurately predict experimentally observed SERS enhancement [60], show the opposite trend, where s25-based BNS particles have a greater maximum heat losses value compared to s50 based BNS particles. While the maximum electric field enhancement value is greater for the top-performing s50-based BNS model, a relatively high degree of electric field enhancement occurs across a much larger portion of the s25 model (Fig. 3E), which theoretically would enhance the SERS signals of a greater number of molecules, leading to an overall greater SERS signal. FEM tools like COMSOL can facilitate the rapid analysis of a wide variety of nanoparticle morphologies to guide synthesis parameter selection to quickly produce particles optimal for a desired application.

### 3.4 Automated Synthesis of Bimetallic Nanostars

**Figure 4-:**
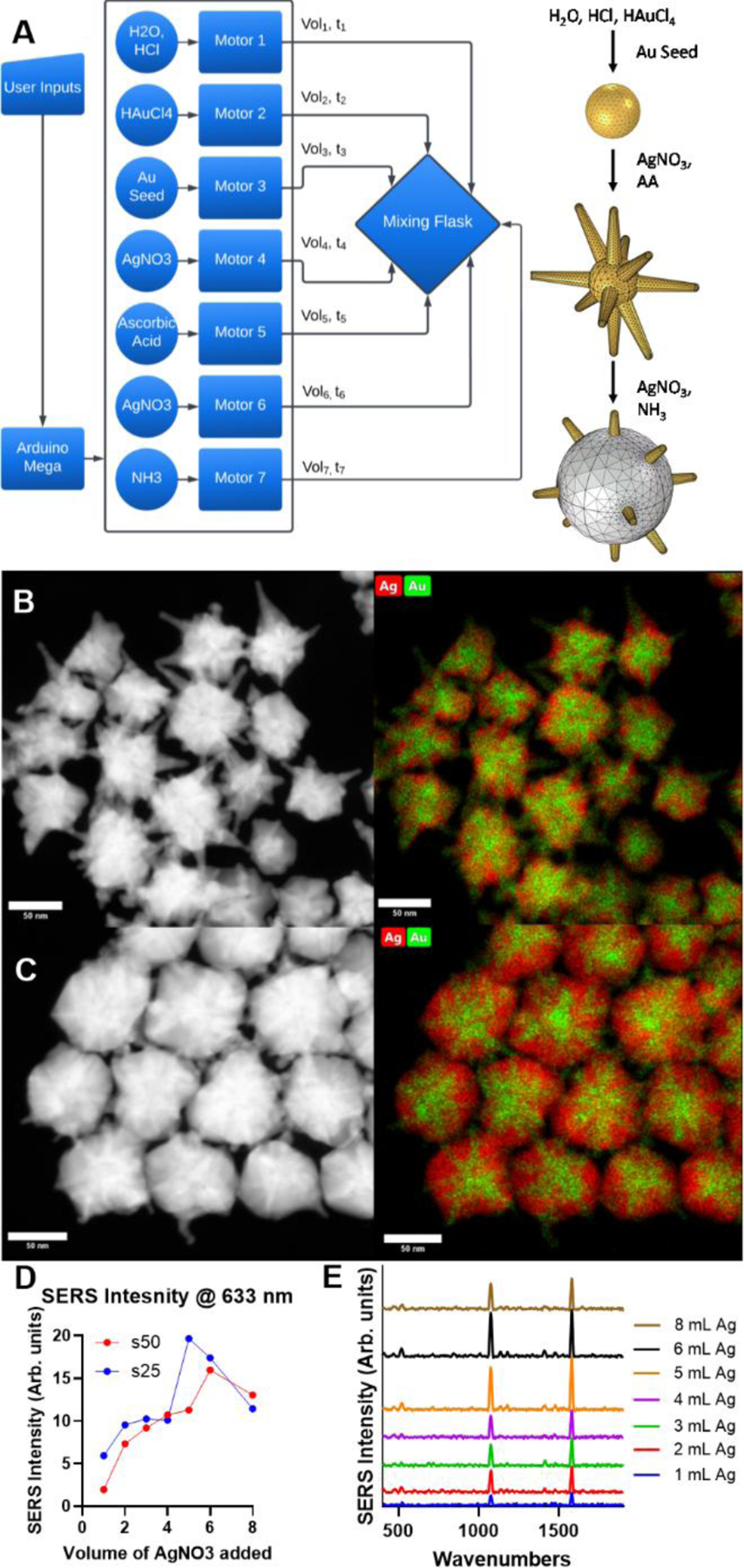
Automated Synthesis and performance evaluation of BNS particles. A) (Left) Flow diagram depicting all of the reagents necessary to synthesize BNS particles. (Right) Schematic of BNS particle formation. B) (Left) HAADF-STEM image and (Right) STEM-EDS image of s50-based BNS particles prepared using 2 mL of silver nitrate. C) (Left) HAADF-STEM image and (Right) STEM-EDS image of s50-based BNS particles prepared using 6 mL of silver nitrate. D) Peak heigh at 1585^−cm^ for different BNS formulations coated with PMBA. E) SERS spectra of s25-based BNS particles coated with p-MBA.

**Figure 5.**
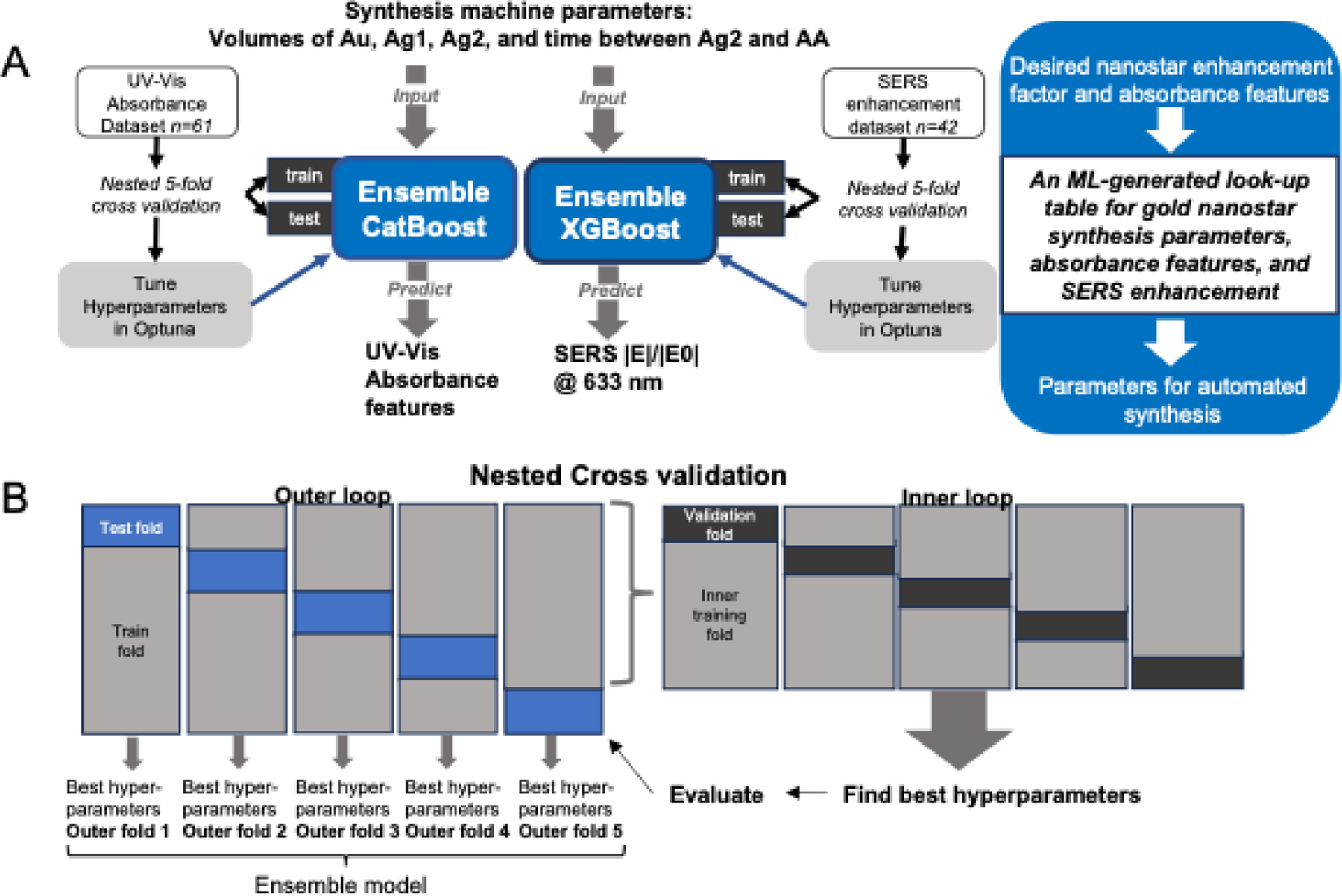
Machine learning augmentation and approach. A) Diagram of machine learning scheme. B) Diagram of 5-fold nested cross validation used to compare the performance of SVR, ANN, RF, XGBoost, and CatBoost for predicting Absorbance characteristics and SERS enhancement.

**Figure 6.**
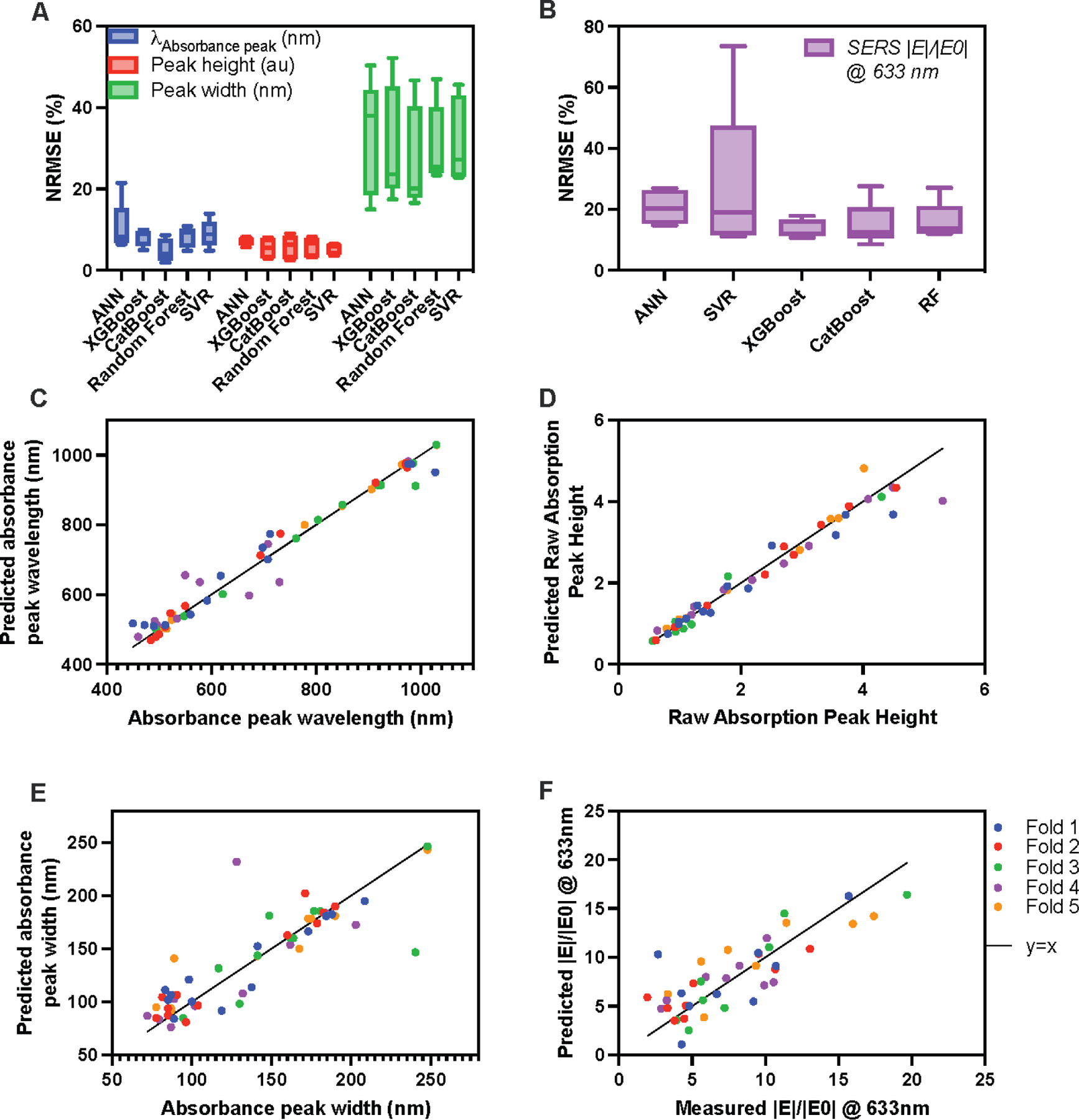
ML Model performance analysis. A) NRMSE results from nested cross validation to predict absorption spectra parameters and (B) SERS enhancement. C) Predicted vs. experimental value of absorbance peak wavelength, (D) raw absorption peak height, (E) absorbance peak width at 85% of normalized peak, and (F) SERS enhancement. The R^2^ fit of data against the perfect x=y prediction line is plotted in red. Predictions from each fold are shown with different shaped markers.

To test the predicted simulated results and demonstrate the modularity and flexibility of this automated synthesis platform, the number of controllable motors was increased by 1 to synthesize BNS particles (Fig. 4A). For these particles, the underlying chemistry shares many similarities with GNS synthesis; however, rather than adding SH-PEG_5000_-COOH as the final step of the reaction, a second addition of silver nitrate occurs, followed by a diluted ammonium hydroxide solution. These differences cause a layer of silver to form on the core of the GNS particle and grow outwards to the nanoparticle branch tips. The s25 and s50 base nanostar morphologies were considered due to their opposing changes in peak absorbance as the amount of gold per particle increased and FEM results. For both the S25 and S50 base particles, 3 mL, 4 mL, and 5 mL of gold chloride was used, and the amount of silver used to coat the different GNS formulations ranged from 1 mL to 8 mL of 12.5 mM silver nitrate solution, resulting in a total of 42 different BNS formulations. The clear trend that emerged in the changes in absorption spectra (Fig. S4A–S4F) as the thickness of the silver layer increased on the GNS surface was the peak absorption value blue shifted and increased. HAADF-STEM and corresponding Energy dispersive X-ray spectroscopy (EDS) were used to observe the morphological differences caused by increasing amounts of silver nitrate in the nanoparticle reaction. For example, as the amount of silver nitrated added to a s50 based particles was increased from 2 mL (Fig. 4B) to 6 mL (Fig. 4C), the thickness of the silver layer substantially increased, growing outwards from the nanoparticle core.

To assess the performance of the different BNS particle formulations for potential use in SERS sensing applications, p-mercaptobenzoic acid (p-MBA) solution was added to aliquots from each batch for a final concentration of 10 **μ**M. The SERS intensity at The SERS spectra of the 42 different BNS formulations was recorded, and the peak height at 1585^−cm^ for both the s25 and s50-based particles when prepared with 5 mL of gold chloride (Fig. 4D) was greater than when either formulation was prepared with 3 mL or 4 mL of gold chloride (Fig. S5A–S5B). The SERS intensity as a function of silver thickness for the s25 and s50 particles closely matches the simulation results (Fig. 3D), where the greatest enhancement is seen with the s25-based particle, at approximately 70% of the maximum amount of silver (Fig. 4E). Further, the maximum SERS intensity generated by s50-based particles occurs at a greater silver amount than with the s25-based particles. The strong agreement between the experimental and simulated morphology-dependent plasmonic properties of nanostar particles both provide validation of the results as well as highlights the potential to further utilize computational tools to investigate a large number of nanoparticle designs and then synthesize them accurately and reproducibly with an automated platform. With the flexibility and repeatability offered by the automated synthesis platform, BNS particles may be finely tuned to maximize SERS enhancement for future incorporation into small molecule and biosensing assays.

### 3.5 Machine learning-assisted synthesis predictions

Although the automated synthesis machine allows precise control of synthesis, experimental optimization of synthesis parameters for specific applications such as photothermal therapy or in-vitro sensing is costly in terms of resources and time intensive. ML predictions of the parameter space based on sparse training data can dramatically reduce the experimental cost of optimization for a variety of applications. A two-ML model workflow was adopted to complete the tasks of 1) predicting absorption spectra features and 2) predicting SERS enhancement by constructing a complete ML-generated look-up table for nanostar particles (Fig 5A). Model inputs consisted of parameters for automated machine during synthesis: volumes of Au, Ag1, Ag2, and time between Ag2 and ascorbic acid addition. These experimental parameters were chosen since they were previously found to greatly influence the optical and SERS properties of resulting synthesized GNS[12]. Separate models were used due to the different datasets available for training. 5-fold nested cross-validation was used for each task to compare the performance of different types of ML models, including support vector regression (SVR), random forest (RF), artificial neural network (ANN), extreme gradient boost (XGBoost), and categorical boost (CatBoost).

Nested cross-validation utilizes two folds of cross-validation and performs hyperparameter tuning within the inner fold cross-validation. Then, the best set of hyperparameters determined from inner fold tuning is trained on all data from the inner folds before evaluating the withheld outer fold. This is repeated for each outer fold (Fig. 5B). Due to limited data, the best hyperparameters found from each outer fold were joined in an ensemble model by averaging their predictions (Table S1–S10). Prediction loss was assessed by normalized root-mean-squared-error (NRMSE) (Eq S1), where NRMSE loss is reported as a percent and defined as RMSE normalized by the range of y values, where n is the number of samples.

In task 1, all models were used to predict the peak absorbance value, the intensity of the peak absorbance value, and the width of the absorbance peak. The highest NRMSE occurred for all models when predicting absorbance peak widths, indicating it is a harder predictive task (Fig. 6A). Most models performed comparably in predicting peak height and width. However, greater variance in NRMSE values was seen between models when predicting the wavelength of absorbance peak. ANN overfit in one outer fold, resulting in a skewed plot and higher standard deviation. ANN is particularly prone to overfitting due to the very large number of parameters in the model. CatBoost performed best overall in task 1: predicting absorbance features from synthesis machine parameters (Fig. 6A). For task 2 validation, the performance of all 5 models at predicting the SERS enhancement of a given formulation from synthesis machine parameters was compared using 5-fold cross-validation. Although the average NRMSE for each model was comparable, SVR had the highest standard deviation due to very high loss only from outer fold 5, pointing to overfitting in outer fold 5 (Fig. 6B). XGBoost NRMSE had the lowest standard deviation between folds and the lowest average and was chosen for the final model. The R^2^ values between predicted and experimental values of each outer fold validation for predicting absorbance peak wavelength, peak height, peak width, and SERS intensity (Fig 6C–6F) were 0.9876, 0.9593, 0.8722, and 0.6641, respectively.

Due to the limited training data, the outer fold loss during nested cross-validation was used to evaluate the model performances. This maximizes the amount of training data available while providing a good estimate of model performance since the outer fold is withheld completely during hyperparameter tuning of the inner folds. CatBoost for task 1 predicted absorbance wavelength and peak height with NRMSE <6%, while peak width had slightly worse accuracy with a loss of 27.2%. XGBoost for task 2 predicted enhancement factors with 633nm excitation wavelengths with NRMSE = 13.94.

**Figure 7.**
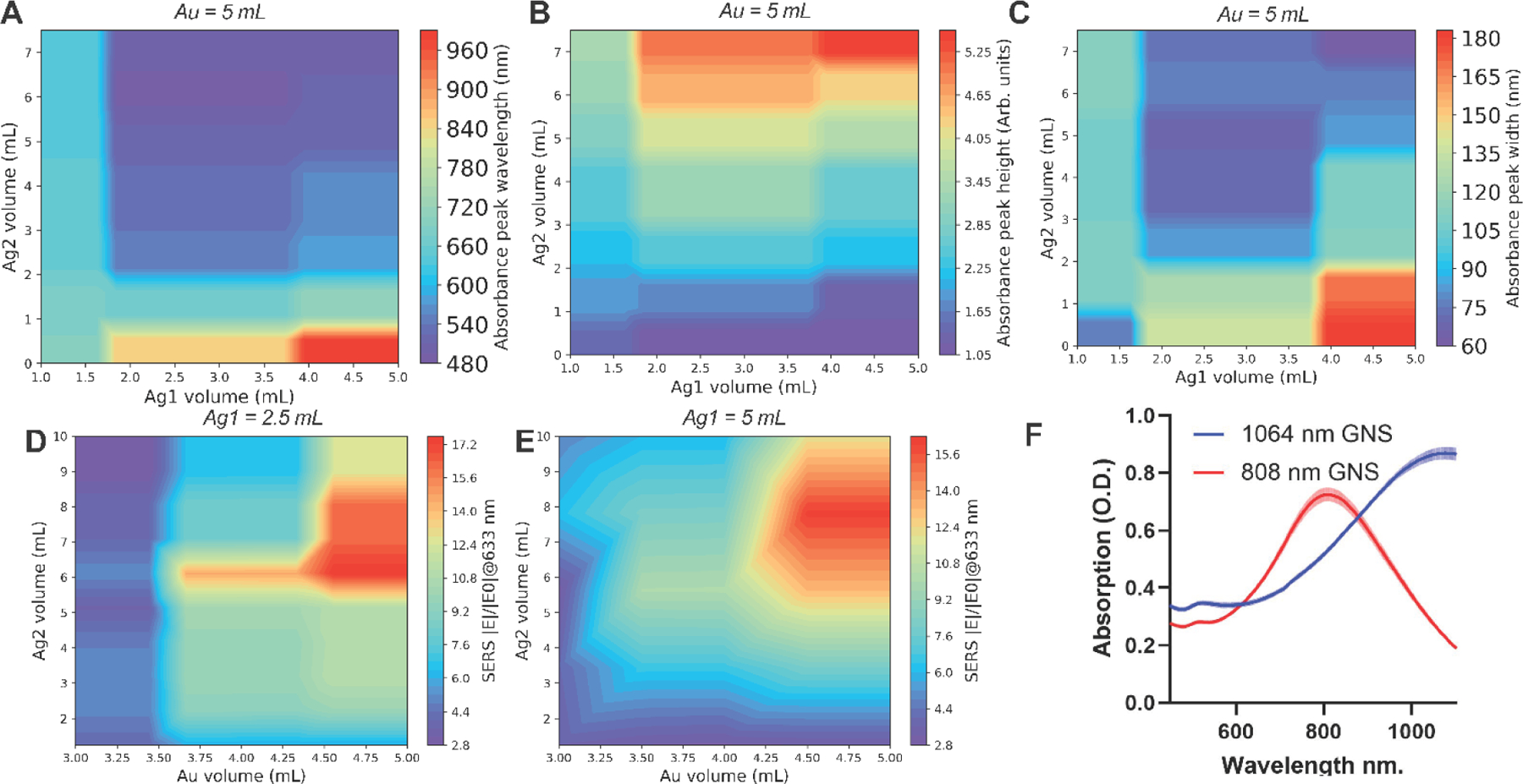
Contour plots of ML predictions. A) Contour plot based on CatBoost predictions for absorbance peak wavelength, (B) absorbance peak width at 85% of normalized peak and (C) height of raw absorbance peak (C). D) Contour plots of XGBoost predictions of SERS enhancement excitation where Ag1 = 2.5 mL and (E) Ag1 = 5 mL. F) Absorption spectra of 808 nm and 1064 nm optimized nanostars, N=6.

These models were then used to construct a look-up table of synthesis parameters, predicted absorbance features, and SERS enhancement. A mesh grid of synthesis parameters for the automated synthesis machine was created to sample the entire parameter space as inputs to both ML models. Given that the volume of the first silver nitrate solution added to the reaction (Ag1) volume was above 1.75 mL, the predicted maximum absorbance peak wavelength quickly blue shifted (decreased) with increasing the volume of the second silver nitrate solution added to the reaction mixture (Ag2) (Fig. 7A), especially at Ag2 = 2mL, which is consistent with previous experimental findings.[41] The highest wavelength absorbance wavelengths occurred around Ag1 >3.75 mL and Ag2 = 0 mL. The predicted absorbance peak height increased with increasing Ag2 volume (Fig. 7B). The predicted absorbance peak widths followed a similar pattern to predicted peak wavelengths (Fig. 7C), as red-shifted absorbance peaks tend to have broader spreads. When Ag1 = 2.5 mL or 5 mL, the SERS enhancement was predicted to be maximum when Ag2 volume was 6–8.5 mL and Au volume was between 4.5–5 mL (Fig. 7D–7E). To validate these lookup tables, we aimed to synthesize gold nanostars with peak absorbance values at two clinically relevant wavelengths for photothermal therapy: 808 nm and 1064 nm. Each prescribed formulation was synthesized 6 times (Fig. 7F). The average peak absorption wavelength for the 808 nm optimized nanostars was 808.3 with a standard deviation of only 4.95 nm or 0.6%, resulting in a percent difference of only 0.04%. The average absorption wavelength for the 1064 nm optimized nanostars was 1076.3 nm with a standard deviation of 2.69 nm or 0.25% and a percent difference of 1.15% from the target wavelength. The excellent performance of this ML-integrated synthetic method highlights the potential of this automated synthesis platform to robustly and reproducibly prepare nanostar particles optimized for specific applications.

## 4. Conclusion

Nanostar particles have excellent, tunable optical properties governed by their branched morphology that induce exceptional plasmonic properties. However, their implementation has been severely limited due to either cytotoxic ionic surfactants used in their synthesis or high batch-to-batch variability inherent in surfactant-free synthetic schemes [61]. This work describes a machine learning-integrated automated synthesis platform that can reproducibly synthesize large volumes of gold nanostar and bimetallic nanostar nanoparticles for a wide array of potential applications constructed with low-cost consumer electronics. This platform will enable the investigation of nanostar particle-based applications, such as photothermal therapy of solid tumors in large animal models previously inhibited by the large amounts of nanoparticles required for such an investigation. Alternatively, the large-scale production of nanostar particle-based SERS substrates can be incorporated into or used to augment current small molecule and biosensing applications. In addition to synthesizing silver-coated gold nanostars, we believe this platform could be adapted to synthesize other high-aspect-ratio bimetallic nanoplatforms like platinum-coated gold nanostars [62], Rhodium-coated nanostars [63], gold-copper alloy nanostars [64], palladium-coated gold nanostars [65], Silver core- gold nanostars [66], and iron-core gold nanostars [67], potentially impacting a wide variety of fields. Using inexpensive components allows rapid adoption of nanostar synthesis and subsequent widespread applications that are otherwise not possible for low-resource laboratories. The small form factor of this synthesis device may even enable the point-of-need synthesis of nanostars in either therapeutic or diagnostic settings. This automated synthesis system with AI capability can produce multifunctional nanostar platforms and has the potential to enable the next phase of investigation for nanostar-based technologies.

## Supplementary Material

Supporting Information
