# Supplementary material for: Tunable and scalable production of nanostar particle platforms for diverse applications using an AI-integrated automated synthesis system
Source: J Mater Sci. Author manuscript; Available in PMC 2026 Jan 7. (PMC12774457; doi:10.1007/s10853-025-10692-1)
Supplement: Supporting Information [file NIHMS2126898-supplement-Supporting_Information.docx]

**Tunable and scalable production of nanostar particle platforms for diverse applications using an AI-integrated automated synthesis system**

Aidan J. Canning ^a,b^, Joy Q. Li ^a,b^, Khang Hoang ^a,b^, Taylor Thorsen ^b,c^, Jianing Chen ^a,b^, Alex Vaziri ^a,b^, Tuan Vo-Dinh ^a,b,c^ *

^a^ Department of Biomedical Engineering, Duke University, Durham, NC, 27708, USA

^b^ Fitzpatrick Institute for Photonics, Duke University, Durham, NC, 27708, USA

^c^ Department of Chemistry, Duke University, Durham, NC, 27708, USA

*Corresponding author- tuan.vodinh@duke.edu

**Supporting Information**

**Figure S1.** Data processing and feature extraction from UV-Vis absorbance.


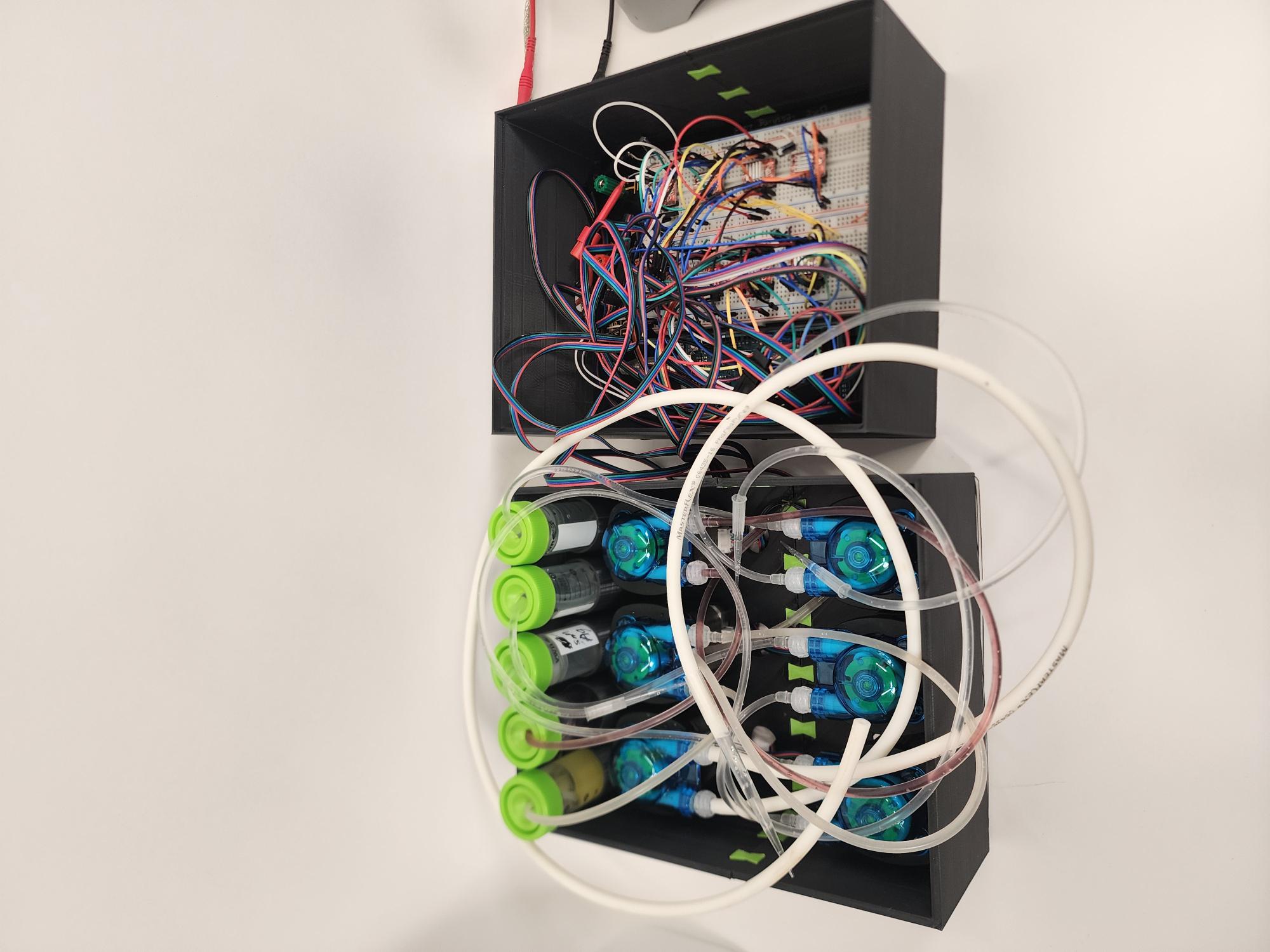


**Figure S2.** All components of the automated synthesis platform are contained within the device housing.


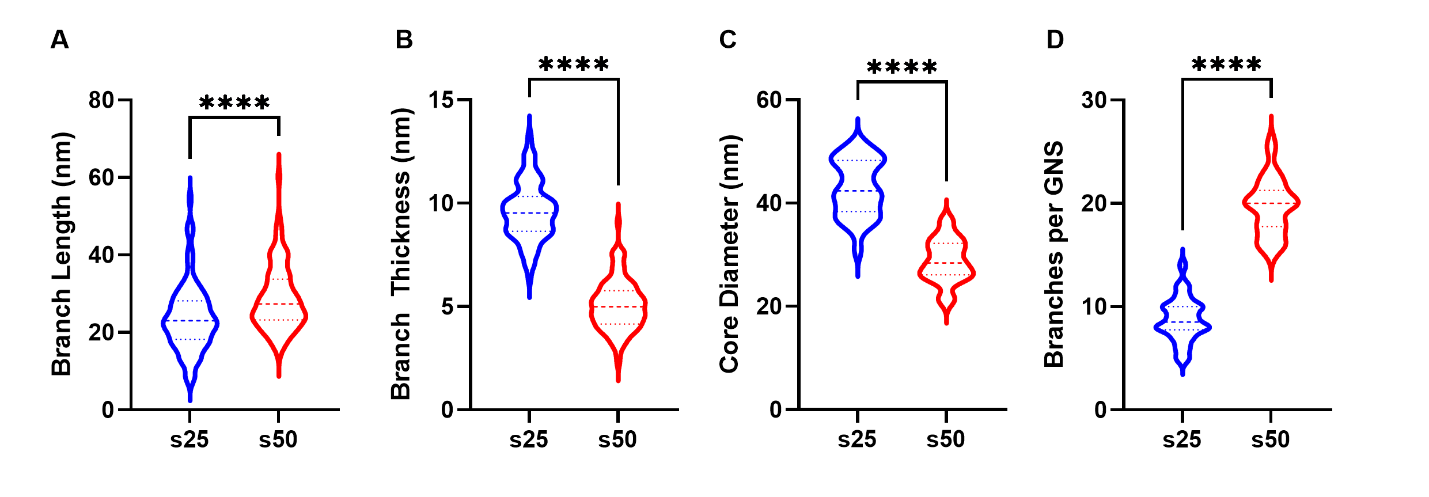


**Figure S3.** Nanoparticle size statistics. A) s25 and s50 branch length measurements (N=100). B) s25 and s50 branch thickness measurements (N=100). C) s25 and s50 core diameter measurements (N=30). D) Branches per particle estimations (N=30).


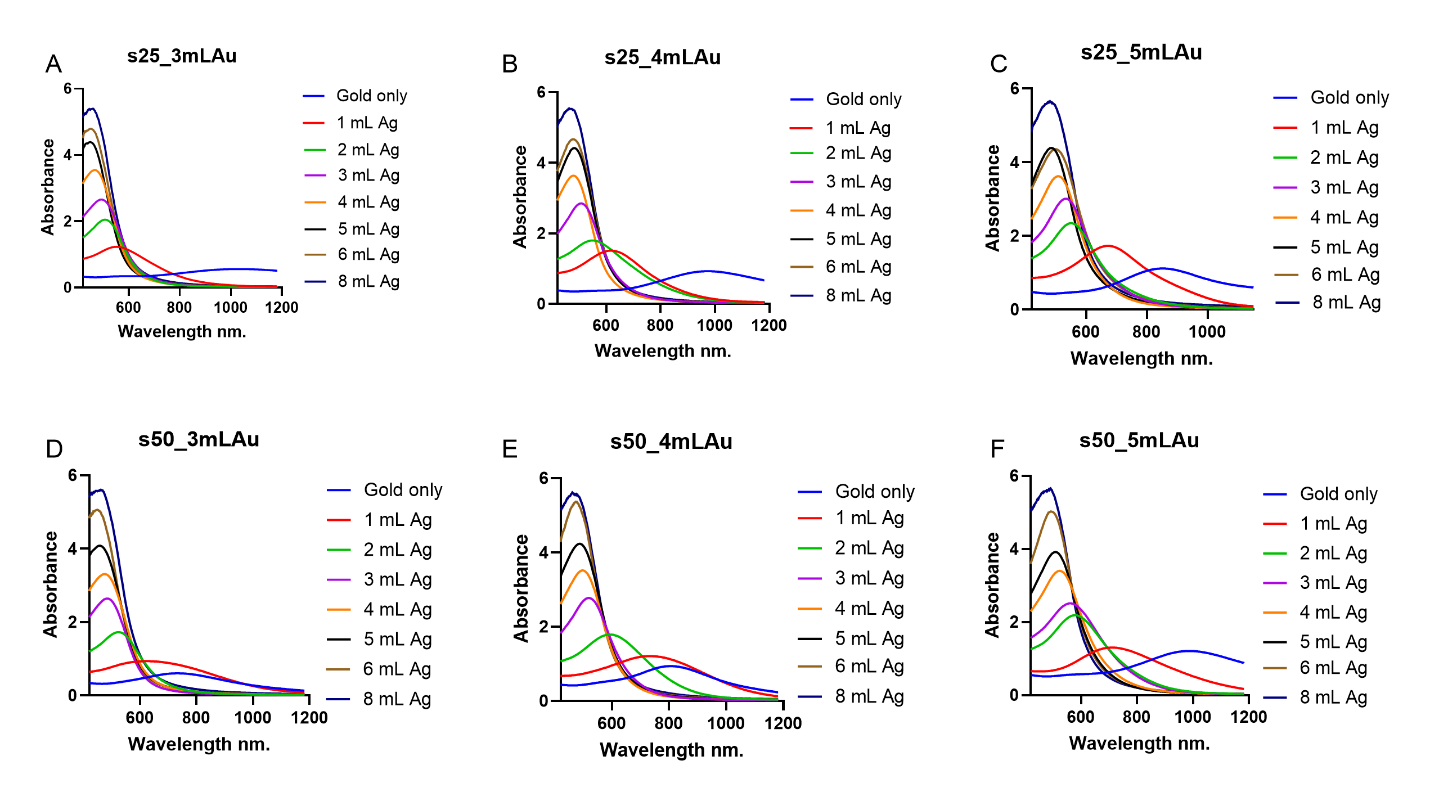


Figure S4. Absorbance spectra of BNS particles for each base GNS morphology as a function of silver. Absorbance spectra of A) s25-Au3 based BNS, B) s25-Au4 based BNS, C) s25-Au5 based BNS, D) s50-Au3 based BNS, E) s50-Au4 based BNS, F) s50-Au5 based BNS.


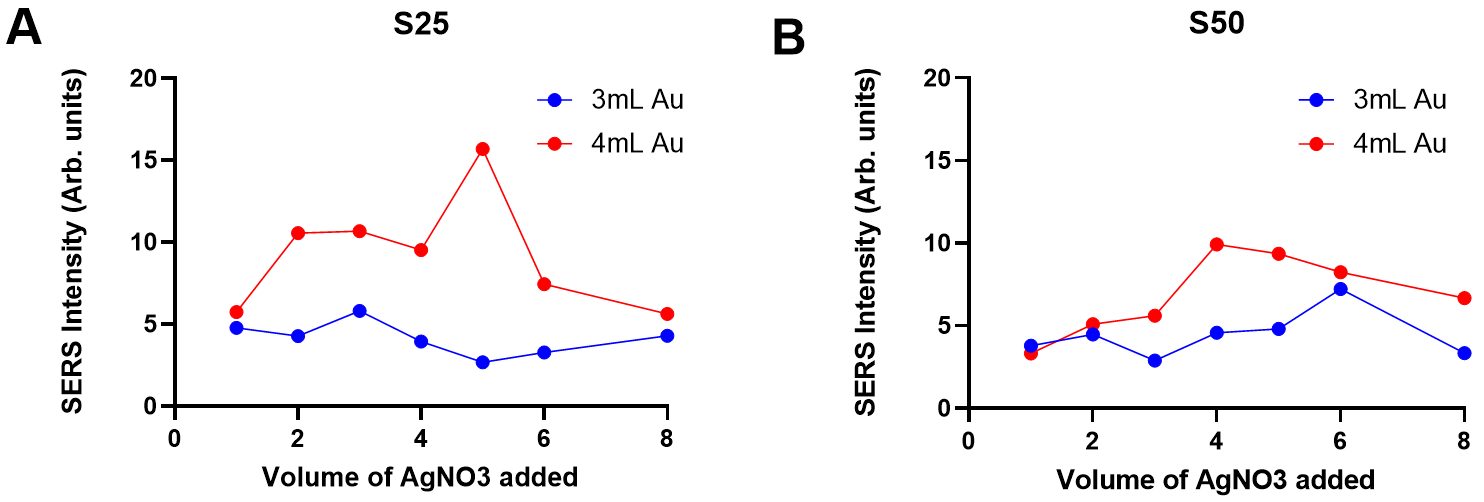


Figure S5. SERS signal at 1076^-cm^ generated by different BNS formulations. A) SERS intensity of S25-Au4 BNS particles at 633nm. B)SERS intensity s50-Au3 based BNS particles at 633nm.

Eq 1. $\left( 1 \right) NRMSE=\frac{\sqrt{\sum_{i=1}^{n} \frac{{(\hat{y}_{i}-y_{i})}^{2}}{n}}}{y_{\max}-y_{\min}}x100$

**Table S1**. CatBoost hyperparameters were tuned for task 1 (absorbance feature prediction) using Optuna n=60 trials for 5-fold nested cross validation. Tuning ranges, layer architecture, and best hyperparameters found were reported for each outer fold.

| Hyperparameter/loss | Variable Type | Range | Tuning Distribution | Best Hyperparameters | | | | |
| --- | --- | --- | --- | --- | --- | --- | --- | --- |
|  |  |  |  | **Fold 1** | **Fold 2** | **Fold 3** | **Fold 4** | **Fold** 5 |
| *Iterations* | Integer | [300, 1000] | uniform | 912 | 975 | 730 | 845 | 451 |
| *Depth* | Integer | [3, 10] | uniform | 9 | 6 | 5 | 3 | 6 |
| *Learning rate* | Continuous | [1x10^-3^, 3x10^-1^] | Log uniform | 5.65x10^-3^ | 3.22x10^-2^ | 2.49x10^-2^ | 2.99x10^-1^ | 2.45x10^-2^ |
| *L2 leaf reg* | Categorical | [1x10^-3^,10] | Log uniform | 3.85x10^-3^ | 1.38x10^-2^ | 1.52x10^-1^ | 3.83x10^-3^ | 1.39x10^-1^ |
| *Random strength* | Continuous | [1x10^-3^,10] | Log uniform | 7.09x10^-1^ | 1.03 | 6.15x10^-1^ | 7.02x10^-3^ | 1.73 |
| *Bagging temperature* | Continuous | [1x10^-3^,10] | Log uniform | 9.64x10^-2^ | 2.25x10^-2^ | 1.79x10^-3^ | 1.61x10^-1^ | 2.79x10^-2^ |
| *Border count* | Integer | [1, 255] | Uniform | 187 | 55 | 155 | 179 | 96 |

**Table S2**. XGBoost hyperparameters were tuned for task 1 (absorbance feature prediction) using Optuna n=60 trials for 5-fold nested cross validation. Tuning ranges, layer architecture, and best hyperparameters found were reported for each outer fold.

| Hyperparameter/loss | Variable Type | Range | Tuning Distribution | Best Hyperparameters | | | | |
| --- | --- | --- | --- | --- | --- | --- | --- | --- |
|  |  |  |  | **Fold 1** | **Fold 2** | **Fold 3** | **Fold 4** | **Fold** 5 |
| *# estimators* | Integer | [100,300] | uniform | 164 | 165 | 226 | 262 | 215 |
| *Max depth* | Categorical | [2,32] | uniform | 8 | 10 | 5 | 8 | 6 |
| *Learning rate* | Continuous | [1x10^-3^, 1x10^-1^] | Log uniform | 7.35x10^-2^ | 9.88x10^-2^ | 8.37x10^-2^ | 7.80x10^-2^ | 6.65x10^-2^ |
| *Subsample* | Continuous | [5x10^-1^, 1] | uniform | 9.73x10^-1^ | 8.43x10^-1^ | 6.21x10^-1^ | 7.26x10^-1^ | 7.95x10^-1^ |
| *Colsample bytree* | Continuous | [5x10^-1^, 1] | uniform | 8.44x10^-1^ | 8.23 x10^-1^ | 9.98x10^-1^ | 9.14x10^-1^ | 9.98x10^-1^ |
| *Gamma* | Continous | [0, 1x10^-1^] | uniform | 8.81x10^-3^ | 3.54x10^-3^ | 5.36x10^-4^ | 9.39x10^-4^ | 7.79x10^-3^ |
| *Lambda* | Continuous | [1x10^-8^, 1] | Log uniform | 8.73x10^-7^ | 2.82x10^-7^ | 1.90x10^-2^ | 3.36x10^-6^ | 8.61x10^-8^ |
| *Alpha* | Continuous | [1x10^-8^, 1x10^-2^] | Log uniform | 3.06x10^-8^ | 1.73x10^-6^ | 1.21x10^-6^ | 1.95x10^-4^ | 2.34x10^-3^ |

**Table S3**. ANN hyperparameters were tuned for task 1 (absorbance feature prediction) using Optuna n=60 trials for 5-fold nested cross validation. Tuning ranges, layer architecture, and best hyperparameters found were reported for each outer fold.

| Hyperparameter/loss | Variable Type | Range | Tuning Distribution | Best Hyperparameters | | | | |
| --- | --- | --- | --- | --- | --- | --- | --- | --- |
|  |  |  |  | **Fold 1** | **Fold 2** | **Fold 3** | **Fold 4** | **Fold** 5 |
| *# hidden layers* | Categorical | [1,3] | uniform | 3 | 1 | 2 | 3 | 2 |
| *# nodes per layer* | Categorical | [2,32] | uniform | 32, 16, 32 | 32 | 9, 16 | 25, 28, 25 | 25, 28, 25 |
| *Learning rate* | Continuous | [1x10^-5^, 1x10^-1^] | Log uniform | 5.40x10^-3^ | 1.60x10^-2^ | 2.01x10^-2^ | 6.40x10^-3^ | 2.26x10^-2^ |
| *Batch size* | Categorical | [8,64] | uniform | 9 | 22 | 43 | 27 | 27 |
| *Dropout* | Continuous | [5x10^-2^, 5x10^-1^] | uniform | 1.48x10^-1^ | 5.05x10^-2^ | 1.99x10^-1^ | 2.32x10^-1^ | 5.06x10^-2^ |
| *Activation* | Categorical | Relu, linear | uniform | relu | relu | linear | linear | linear |

**Table S4**. SVR hyperparameters were tuned for task 1 (absorbance feature prediction) using Optuna n=60 trials for 5-fold nested cross validation. Tuning ranges, layer architecture, and best hyperparameters found were reported for each outer fold.

| Hyperparameter/loss | Variable Type | Range | Tuning Distribution | Best Hyperparameters | | | | |
| --- | --- | --- | --- | --- | --- | --- | --- | --- |
|  |  |  |  | **Fold 1** | **Fold 2** | **Fold 3** | **Fold 4** | **Fold** 5 |
| *kernel* | Categorical | Linear, poly, ref, sigmoid | uniform | rbf | rbf | rbf | rbf | rbf |
| *C* | Continuous | [1x10-2, 1x10^2] | Log uniform | 60.5 | 8.68 | 38.0 | 2.90 | 66.6 |
| *epsilon* | Continuous | [1x10^-4^, 1x10^-2^] | Log uniform | 7.00x10^-2^ | 2.88x10^-4^ | 1.20x10^-2^ | 6.87x10^-3^ | 1.10x10^-3^ |
| *gamma* | Categorical | Scale, auto | uniform | auto | scale | auto | scale | auto |

**Table S5**. RF hyperparameters were tuned for task 1 (absorbance feature prediction) using Optuna n=60 trials for 5-fold nested cross validation. Tuning ranges, layer architecture, and best hyperparameters found were reported for each outer fold.

| Hyperparameter/loss | Variable Type | Range | Tuning Distribution | Best Hyperparameters | | | | |
| --- | --- | --- | --- | --- | --- | --- | --- | --- |
|  |  |  |  | **Fold 1** | **Fold 2** | **Fold 3** | **Fold 4** | **Fold** 5 |
| *# estimators* | categorical | [100, 300] | uniform | 287 | 289 | 119 | 233 | 236 |
| *Max depth* | integer | [3, 20] | uniform | 17 | 13 | 15 | 7 | 19 |
| *Min samples split* | integer | [1, 10] | uniform | 2 | 2 | 2 | 2 | 2 |
| *Min samples leaf* | integer | [1, 10] | uniform | 1 | 1 | 1 | 1 | 1 |
| *Max features* | categorical | Auto, sqrt, log2 | uniform | Log2 | auto | Log2 | auto | auto |

**Table S6**. XGBoost hyperparameters were tuned for task 2 (SERS enhancement prediction) using Optuna n=60 trials for 5-fold nested cross validation. Tuning ranges, layer architecture, and best hyperparameters found were reported for each outer fold.

| Hyperparameter/loss | Variable Type | Range | Tuning Distribution | Best Hyperparameters | | | | |
| --- | --- | --- | --- | --- | --- | --- | --- | --- |
|  |  |  |  | **Fold 1** | **Fold 2** | **Fold 3** | **Fold 4** | **Fold** 5 |
| *# estimators* | Integer | [100,300] | uniform | 300 | 185 | 266 | 102 | 259 |
| *Max depth* | Categorical | [2,32] | uniform | 12 | 11 | 9 | 4 | 12 |
| *Learning rate* | Continuous | [1x10^-3^, 1x10^-1^] | Log uniform | 6.36x10^-2^ | 7.40x10^-2^ | 8.06x10^-2^ | 8.35x10^-2^ | 9.80x10^-2^ |
| *Subsample* | Continuous | [5x10^-1^, 1] | uniform | 8.30x10^-1^ | 8.37x10^-1^ | 6.86x10^-1^ | 5.04x10^-1^ | 5.16x10^-1^ |
| *Colsample bytree* | Continuous | [5x10^-1^, 1] | uniform | 8.16x10^-1^ | 7.81 x10^-1^ | 9.81x10^-1^ | 9.49x10^-1^ | 8.86x10^-1^ |
| *Gamma* | Continous | [0, 1x10^-1^] | uniform | 8.81x10^-3^ | 4.01x10^-3^ | 4.44x10^-3^ | 3.16x10^-4^ | 4.11x10^-3^ |
| *Lambda* | Continuous | [1x10^-8^, 1] | Log uniform | 9.91x10^-3^ | 2.47x10^-7^ | 1.86x10^-7^ | 8.38x10^-8^ | 5.02x10^-3^ |
| *Alpha* | Continuous | [1x10^-8^, 1x10^-2^] | Log uniform | 1.10x10^-8^ | 1.08x10^-7^ | 1.08x10^-7^ | 6.46x10^-3^ | 3.61x10^-3^ |

**Table S7**. ANN hyperparameters were tuned for task 2 (SERS enhancement prediction) using Optuna n=60 trials for 5-fold nested cross validation. Tuning ranges, layer architecture, and best hyperparameters found were reported for each outer fold.

| Hyperparameter/loss | Variable Type | Range | Tuning Distribution | Best Hyperparameters | | | | |
| --- | --- | --- | --- | --- | --- | --- | --- | --- |
|  |  |  |  | **Fold 1** | **Fold 2** | **Fold 3** | **Fold 4** | **Fold** 5 |
| *# hidden layers* | Categorical | [1,3] | uniform | 3 | 2 | 2 | 2 | 2 |
| *# nodes per layer* | Categorical | [2,32] | uniform | 2, 9, 28 | 5, 30 | 28, 21 | 24, 30 | 30, 26 |
| *Learning rate* | Continuous | [1x10^-5^, 1x10^-1^] | Log uniform | 5.69x10^-4^ | 7.03x10^-4^ | 7.97x10^-4^ | 6.78x10^-4^ | 7.24x10^-4^ |
| *Batch size* | Categorical | [8,64] | uniform | 21 | 24 | 29 | 14 | 22 |
| *Dropout* | Continuous | [5x10^-2^, 5x10^-1^] | uniform | 1.13x10^-1^ | 1.01x10^-1^ | 1.68x10^-1^ | 1.35x10^-1^ | 2.22x10^-1^ |
| *Activation* | Categorical | Relu, linear | uniform | linear | relu | relu | relu | relu |

**Table S8**. SVR hyperparameters were tuned for task 2 (SERS enhancement prediction) using Optuna n=60 trials for 5-fold nested cross validation. Tuning ranges, layer architecture, and best hyperparameters found were reported for each outer fold.

| Hyperparameter/loss | Variable Type | Range | Tuning Distribution | Best Hyperparameters | | | | |
| --- | --- | --- | --- | --- | --- | --- | --- | --- |
|  |  |  |  | **Fold 1** | **Fold 2** | **Fold 3** | **Fold 4** | **Fold** 5 |
| *kernel* | Categorical | Linear, poly, ref, sigmoid | uniform | Poly, degree 2 | rbf | Poly, degree 2 | linear | rbf |
| *C* | Continuous | [1x10^-2^, 1x10^2^] | Log uniform | 977 | 2.17 | 984 | 23.5 | 196 |
| *epsilon* | Continuous | [1x10^-4^, 1x10^-2^] | Log uniform | 1.34x10^-1^ | 9.59x10^-2^ | 1.88x10^-3^ | 8.10x10^-2^ | 8.10x10^-2^ |

**Table S9**. RF hyperparameters were tuned for task 2 (SERS enhancement prediction) using Optuna n=60 trials for 5-fold nested cross validation. Tuning ranges, layer architecture, and best hyperparameters found were reported for each outer fold.

| Hyperparameter/loss | Variable Type | Range | Tuning Distribution | Best Hyperparameters | | | | |
| --- | --- | --- | --- | --- | --- | --- | --- | --- |
|  |  |  |  | **Fold 1** | **Fold 2** | **Fold 3** | **Fold 4** | **Fold** 5 |
| *# estimators* | categorical | [100, 300] | uniform | 90 | 281 | 204 | 141 | 202 |
| *Max depth* | integer | [3, 20] | uniform | 16 | 15 | 13 | 16 | 19 |
| *Min samples split* | integer | [1, 10] | uniform | 3 | 2 | 2 | 2 | 3 |
| *Min samples leaf* | integer | [1, 10] | uniform | 1 | 2 | 1 | 1 | 2 |
| *Max features* | categorical | Auto, sqrt, log2 | uniform | auto | None | None | None | None |

**Table S10**. CatBoost hyperparameters were tuned for task 2 (SERS enhancement prediction) using Optuna n=60 trials for 5-fold nested cross validation. Tuning ranges, layer architecture, and best hyperparameters found were reported for each outer fold.

| Hyperparameter/loss | Variable Type | Range | Tuning Distribution | Best Hyperparameters | | | | |
| --- | --- | --- | --- | --- | --- | --- | --- | --- |
|  |  |  |  | **Fold 1** | **Fold 2** | **Fold 3** | **Fold 4** | **Fold 5** |
| *Iterations* | Integer | [300, 1000] | uniform | 336 | 655 | 882 | 994 | 726 |
| *Depth* | Integer | [3, 10] | uniform | 3 | 4 | 3 | 4 | 3 |
| *Learning rate* | Continuous | [1x10^-3^, 3x10^-1^] | Log uniform | 4.41x10^-2^ | 1.46x10^-1^ | 1.97x10^-1^ | 2.16x10^-1^ | 4.26x10^-2^ |
| *L2 leaf reg* | Categorical | [1x10^-3^,10] | Log uniform | 5.57x10^-3^ | 1.09 | 1.94x10^-3^ | 8.62x10^-3^ | 1.12x10^-3^ |
| *Random strength* | Continuous | [1x10^-3^,10] | Log uniform | 2.78 | 3.57 | 6.43 | 8.45 | 7.68x10-1 |
| *Bagging temperature* | Continuous | [1x10^-3^,10] | Log uniform | 8.85 | 8.29x10^-3^ | 3.19 | 1.69x10^-1^ | 1.97x10^-3^ |
| *Border count* | Integer | [1, 255] | Uniform | 109 | 100 | 171 | 245 | 104 |

**Table S11.** For task 1, NRMSE of each model type by fold for absorbance peak wavelength prediction.

| **Model 2** | **Fold 1** | **Fold 2** | **Fold 3** | **Fold 4** | **Fold 5** |
| --- | --- | --- | --- | --- | --- |
| ***ANN*** | 8.93035978 | 21.4833903 | 6.35859114 | 8.23936052 | 7.38460857 |
| ***SVR*** | 8.97177855 | 9.02657567 | 7.16865412 | 9.97405347 | 5.01314136 |
| ***XGBoost*** | 6.67876452 | 3.02797584 | 4.08326124 | 8.64805462 | 1.85924878 |
| ***CatBoost*** | 9.21711314 | 10.8050495 | 6.5761295 | 9.74300445 | 4.84300443 |
| ***RF*** | 9.84541778 | 4.82600297 | 8.89332833 | 13.9052723 | 7.6859129 |

**Table S12.** For task 1, NRMSE of each model type by fold for absorbance peak height prediction.

| **Model 2** | **Fold 1** | **Fold 2** | **Fold 3** | **Fold 4** | **Fold 5** |
| --- | --- | --- | --- | --- | --- |
| ***ANN*** | 6.53528361 | 8.15171454 | 7.74764102 | 7.65166654 | 5.68310879 |
| ***SVR*** | 5.61789531 | 2.88766158 | 2.99529487 | 7.7613122 | 8.06928767 |
| ***XGBoost*** | 6.28452373 | 2.54947183 | 3.2049892 | 8.07497487 | 9.01384191 |
| ***CatBoost*** | 6.65557548 | 3.38767126 | 3.18190082 | 8.15925432 | 7.47687436 |
| ***RF*** | 6.47636612 | 3.60539142 | 4.15627952 | 6.41813663 | 6.13905558 |

**Table S13.** For task 1, NRMSE of each model type by fold for absorbance peak width prediction.

| **Model 2** | **Fold 1** | **Fold 2** | **Fold 3** | **Fold 4** | **Fold 5** |
| --- | --- | --- | --- | --- | --- |
| ***ANN*** | 14.9690698 | 50.3140203 | 37.9825615 | 38.209777 | 22.0846618 |
| ***SVR*** | 22.8179656 | 23.6370201 | 37.9406293 | 52.1668961 | 17.4403103 |
| ***XGBoost*** | 19.1557463 | 16.5771664 | 33.6107503 | 46.6944814 | 20.1168776 |
| ***CatBoost*** | 24.7521872 | 25.4198289 | 33.1240755 | 46.9555938 | 23.3749742 |
| ***RF*** | 22.8366625 | 23.3669041 | 40.3330396 | 45.5872392 | 27.2056108 |

**Table S14.** For task 2, NRMSE of each model type by fold for |E|/|Eo| @ 633nm excitation prediction.

| **Model 2** | **Fold 1** | **Fold 2** | **Fold 3** | **Fold 4** | **Fold 5** |
| --- | --- | --- | --- | --- | --- |
| ***ANN*** | 15.778264 | 25.6477762 | 20.2646535 | 14.8084884 | 26.9653573 |
| ***SVR*** | 21.0973956 | 12.1689047 | 19.0419881 | 11.1760226 | 73.5316097 |
| ***XGBoost*** | 17.8923813 | 10.7600796 | 11.9867777 | 11.8097268 | 15.48544 |
| ***CatBoost*** | 13.6719808 | 12.5091756 | 8.55368314 | 12.4360793 | 27.5406301 |
| ***RF*** | 14.9289672 | 12.0525373 | 13.8598762 | 12.3674094 | 27.1122918 |
